# The association between environmental exposures during childhood and the subsequent development of Crohn’s disease: A score analysis approach

**DOI:** 10.1371/journal.pone.0171742

**Published:** 2017-02-07

**Authors:** Victor Tinashe Sabe, Abigail Raffner Basson, Esme Jordaan, Mikateko Mazinu

**Affiliations:** 1 Medical BioScience Department, University of the Western Cape, Bellville, Western Cape, South Africa; 2 Division of Gastroenterology and Liver Disease, Cominelli Laboratory, School of Medicine, Case Western Reserve University, Cleveland, Ohio, United States of America; 3 Biostatistics Unit, Medical Research Council of South Africa, Parow, Western Cape, South Africa; 4 Biostatistics Unit, Medical Research Council of South Africa, Parow, Western Cape, South Africa and the Statistics and Population Studies Department, University of the Western Cape, Bellville, Western Cape, South Africa; Children's Hospital of Los Angeles, UNITED STATES

## Abstract

**Background:**

Environmental factors during childhood are thought to play a role in the aetiology of Crohn’s Disease (CD). In South Africa, recently published work based on an investigation of 14 childhood environmental exposures during 3 age intervals (0–5, 6–10 and 11–18 years) has provided insight into the role of timing of exposure in the future development of CD. The ‘overlapping’ contribution of the investigated variables however, remains unclear. The aim of this study was to perform a post hoc analysis using this data and investigate the extent to which each variable contributes to the subsequent development of CD relative to each aforementioned age interval, based on a score analysis approach.

**Methods:**

Three methods were used for the score analysis. Two methods employed the subgrouping of one or more (similar) variables (methods A and B), with each subgroup assigned a score value weighting equal to one. For comparison, the third approach (method 0) involved no grouping of the 14 variables. Thus, each variable held a score value of one.

**Results:**

Results of the score analysis (Method 0) for the environmental exposures during 3 age intervals (0–5, 6–10 and 11–18 years) revealed no significant difference between the case and control groups. By contrast, results from Method A and Method B revealed a significant difference during all 3 age intervals between the case and control groups, with cases having significantly lower exposure scores (approximately 30% and 40% lower, respectively).

**Conclusion:**

Results from the score analysis provide insight into the ‘compound’ effects from multiple environmental exposures in the aetiology of CD.

## Introduction

Environmental risk factors in childhood are believed to play a role in the subsequent development of the inflammatory bowel disease (IBD) subtype, Crohn’s disease (CD) [1–4]. Numerous studies have evaluated the different environmental exposures during childhood, albeit findings for many have been inconsistent [4–14]. These inconsistencies may be attributed to differences in both the timing and the extent of the various environmental exposures during childhood, as well as the heterogeneity in CD susceptibility mutations both between and within individual population groups [15, 16]. Alternatively, these findings may be a result of methodological issues such as study sample size, participant characteristics (i.e. demographics, socioeconomic factors), identification of inappropriate control subjects, or the failure to account for potential confounding environmental variables.

Several theories have emanated in a bid to understand the underlying pathogenesis and aetiology of IBD [17–20]. The most widely accepted is the ‘hygiene hypothesis’ and the theory has received wide speculation and academic attention [19]. The hygiene hypothesis holds that an ‘overly hygienic’ childhood environment will impair the microbial competence of the gastrointestinal immune system, and its ability to appropriately recognize new antigens, predisposing children to immunologic disorders later in life [19, 21]. While the effect is deemed to be most profound during early childhood, the optimal timing and magnitude of exposure required is still unclear. In addition, it is entirely possible that the impact of different exposures is not mutually exclusive; that disease development depends on the dose-response interactions between exposures, and this reflects how strongly one exposure may ‘protect’ or increase the ‘risk’ conferred by CD susceptibility mutations. The first of many susceptibility genes identified was CARD15 (caspase-activation recruitment domain), also referred to as the NOD2 (nucleotide oligomerization domain) gene, within the IBD1 (inflammatory bowel disease) locus. The gene is responsible for the recognition and clearance of intestinal antigens via the induction of autophagy-related protein complexes. Polymorphisms in the CARD15/NOD2/IBD1 locus have been associated with the highest risk for CD development [22–24] and recent evidence shows reduced functionality affects the immune system and disease phenotype in paediatric CD population [25]. Indeed the role of epigenetics and alterations in DNA methylation in IBD cannot be ignored [26]. The concept of microbiota or environmentally-induced permanent epigenetic change particularly within developing areas is a distinct possibility [27].

Numerous studies have been conducted on the paradigm of the hygiene hypothesis, including the recent case control investigation of childhood environmental exposures (0–5, 6–10 and 11–18 years) conducted in the 2 largest tertiary referral IBD centres in Cape Town, South Africa by Basson et al [4]. It must be highlighted that, more recent literature denotes distinct differences between adult and paediatric IBD patient population, particularly with regard to matters surrounding the hygiene hypothesis [28]. The distinction between these two groups has been recently debated and it appears that with IBD are a distinctive population with specific peculiarities requiring highly skilled and specialised approach for diagnosis and treatment [28].

The present study uses a subset of data from the previously mentioned study by Basson et al [4]. The aim of the present study was to investigate the association between childhood environmental exposures and the risk of CD development in the Western Cape, South Africa, based on a score analysis approach.

## Materials and methods

### Design and setting

This was a post hoc analysis of previously collected data from a large case-control study, performed between September 2011 and January 2013, of all consecutive CD patients seen during their normally scheduled appointments at the 2 largest public sector hospitals in Cape Town; Groote Schuur Hospital (GSH) and Tygerberg Hospital (TBH). The methodological approaches of the study have been described elsewhere [4]. Disease characteristics of the CD patients have also been described in detail elsewhere [29]. Briefly, only patients with complete data at diagnosis were included. Patients were excluded if disease duration was less than 5 years, or had a prior diagnosis of intestinal tuberculosis, determined via the algorithm suggested by Epstein et al [30]. Control subjects were identified from the same populations giving rise to the CD cases. Controls were excluded if they had a prior diagnosis of tuberculosis, IBD or other immune-mediated diseases, any gastrointestinal disorder (e.g., irritable bowel syndrome), or any family history of IBD.

### Study data

For reference, the results from Basson et al of the multiple logistic regression analysis evaluating environmental risk factor exposure in 3 age groups (0–5 years, 6–10 years and 11–18 years) have been presented in Table 1. The authors investigated 14 environmental exposures, including; primary source of drinking water, hot piped tap water, community type, total number of people in household, number of people sharing a bathroom, number of bedrooms in home, type of toilet facility, type of toilet facility, donkey/horse/cow/sheep living permanently on the property, second-hand cigarette smoke exposure, unpasteurized milk consumption, raw beef consumption, helminth infection and treatment for helminths. The relevant data is available via the link: http://dx.doi.org/10.6084/m9.figshare.1041586.

**Table 1:** Environmental risk factors over three age intervals; 0–5 years, 6–10 years and 11–18 years. [4].

	0–5 years			6–10 years			11–18 years		
	Cases n (%)	Control n (%)	Adjusted OR (95% CI)*	Cases n (%)	Control n (%)	Adjusted OR (95% CI)*	Cases n (%)	Control n (%)	Adjusted OR (95% CI)*
**Primary source of drinking water**									
Piped or bottled water	162 (84)	172 (80)	**2.10 (1.20, 4.00)**	171 (88)	183 (86)	**2.05 (1.10, 4.10)**	94 (194)	194 (91)	1.92 (0.80, 4.60)
Outside tap/borehole/well/river/dam	31 (16)	42 (20)		23 (12)	31 (14)		6 (19)	19 (9)	
**Hot piped tap water**									
Access	59 (32)	85 (40)	1.18 (0.71, 2.00)	77 (40)	99 (46)	1.52 (0.92, 2.54)	112 (58)	129 (60)	1.52 (0.93, 2.51)
No access	128 (68)	127 (60)		116 (60)	114 (54)		82 (42)	85 (40)	
**Community type**									
Suburban or Urban	136 (76)	146 (72)	1.21 (0.71, 2.10)	147 (80)	156 (74)	1.58 (0.91, 2.76)	152 (80)	168 (80)	0.97 (0.55, 1.70)
Rural or farm or informal settlement	43 (24)	58 (28)		36 (20)	54 (26)		38 (20)	42 (20)	
**Total number of people in household**									
5 or less	77 (41)	123 (59)	0.68 (0.43, 1.09)	79 (41)	130 (62)	0.67 (0.42, 1.06)	100 (52)	129 (61)	1.05 (0.66, 1.69)
6 or more	111 (59)	86 (41)		113 (59)	81 (38)		94 (48)	82 (39)	
**Number of people sharing a bathroom**									
3 or less	31 (17)	59 (29)	**0.55 (0.31, 0.97)**	27 (15)	62 (30)	**0.51 (0.28, 0.90)**	36 (19)	65 (31)	0.65 (0.38, 1.11)
4 or more	151 (83)	147 (71)		159 (85)	146 (70)		157 (81)	147 (69)	
**Number of bedrooms in home**									
3 or more	82 (44)	110 (52)	0.87 (0.69, 1.11)	87 (46)	116 (55)	0.87 (0.69, 1.10)	110 (57)	131 (61)	0.91 (0.72, 1.14)
2 or less	104 (56)	101 (48)		102 (54)	94 (45)		83 (43)	83 (39)	
**Type of toilet facility**									
Flush (own family or shared)	154 (81)	187 (89)	1.35 (0.69, 2.63)	166 (86)	190 (90)	1.62 (0.78, 3.38)	180 (94)	203 (95)	1.76 (0.60, 5.08)
Bucket, pit latrine, no facility	36 (19)	24 (11)		26 (14)	21 (10)		11 (6)	10 (5)	
**Household pets**									
No	95 (51)	81 (39)	1.47 (0.92, 2.34)	91 (48)	96 (45)	1.13 (0.71, 1.79)	88 (46)	102 (48)	1.01 (0.64, 1.59)
Yes	93 (49)	126 (61)		100 (52)	116 (55)		103 (54)	112 (52)	
**Donkey/horse/cow/sheep on property**									
No	172 (90)	179 (85)	1.67 (0.83, 3.45)	178 (94)	179 (84)	**3.10 (1.42, 7.21)**	187 (97)	199 (93)	**4.31 (1.36, 16.14)**
Yes	19 (10)	32 (15)		12 (6)	34 (16)		5 (3)	15 (7)	
**Cigarette smoke exposure**									
Yes	145 (78)	150 (71)	**1.71 (1.01, 2.94)**	149 (78)	158 (74)	1.63 (0.95, 2.83)	155 (80)	149 (70)	**2.03 (1.20, 3.48)**
No	42 (22)	60 (29)		42 (22)	55 (26)		39 (20)	65 (30)	
**Unpasteurized milk consumption**									
Never	169 (96)	139 (78)	**8.02 (3.19, 23.28)**	169 (93)	149 (73)	**6.43 (3.02, 14.81)**	178 (93)	179 (85)	**2.69 (1.23, 6.17)**
Once per year or more	7 (4)	39 (22)		12 (7)	54 (27)		13 (7)	32 (15)	
**Raw beef consumption**									
Never	164 (95)	163 (86)	**2.84 (1.17, 7.56)**	171 (92)	173 (85)	**2.31 (1.00, 5.80)**	174 (92)	184 (86)	1.48 (0.69,3.29)
Once per year or more	9 (5)	27 (14)		11 (96)	29 (15)		15 (8)	29 (14)	
**Helminth infection**									
No	82 (52)	107 (56)	0.87 (0.53, 1.42)	104 (58)	125 (63)	0.85 (0.53, 1.37)	166 (88)	171 (81)	**1.90 (1.00, 3.71)**
Yes	77 (48)	84 (44)		74 (42)	75 (38)		23 (12)	40 (19)	
**Treatment for helminths**									
No	72 (48)	88 (47)	0.99 (0.60, 1.63)	98 (57)	105 (53)	1.17 (0.73, 1.89)	152 (82)	155 (74)	1.70 (0.95, 2.97)
Yes	79 (52)	98 (53)		74 (43)	95 (48)		33 (18)	55 (26)	

*OR odds ratio adjusted for age at study enrolment, ethnicity and gender, and 95% confidence interval. Subjects who responded ‘do not know’ were excluded from analysis. Table Reproduced from BASSON et al [4]

Data for the disease characteristics of the CD patients is available via the link: http://dx.doi.org/10.6084/m9.figshare.1041586.

### Methodology

Environmental Exposures for the age intervals (0–5 years, 6–10 years and 11–18 years). To evaluate the environmental exposures during the 3 age intervals based on a score analysis of the 14 environmental exposure variables (Table 1), three different methodological approaches were undertaken. In the first approach (‘Method 0’), no score value was assigned to the variables, thus all 14 variables had a weighting score value equal to one. In the second and third analytical approach, environmental variables of similar nature were combined into ‘subgroups’ based on two methodological approaches termed ‘Method A’ and ‘Method B’. All 14 variables were included in the two approaches (Method A, Method B), and only the number of subgroups within Method A and Method B differed. Each ‘subgroup’ was equal to a value of one, and relative to the methodology for variable subgrouping, the weighting of an environmental variable was inversely related to the number of variables contained within its respective subgroup. For instance, within the ‘Method A’ approach, the environmental exposure variables ‘water source’ and ‘hot water availability’ were grouped together (i.e. subgroup A1), to form one subgroup, whereas the exposure variables ‘number of people in the household’, ‘number of bedrooms in the household’ and ‘pets in the home’ were combined to form another subgroup (i.e. subgroup A2). Both subgroup A1 and subgroup A2 had a score value equal to a value of one, respectively. However the individual weighting for the two variables comprised within subgroup A1 (‘water source’ and ‘hot water’) was 1/2 + 1/2, whereas for the three variables within subgroup A2 (‘number of people in the household’, ‘number of bedrooms in the household’ and ‘pets in the home’) was 1/3 + 1/3 + 1/3. The subgrouping of variables and the weighted score of variable(s) within each subgroup for Method A (outcome 1) and of Method B (outcome 2) are depicted in Tables 2 and 3, respectively.

**Table 2:** Score analysis for Method A.

Environmental Risk Factor Combination	Weighting for each variable	Total score for subgroup
Source of drinking water (Not piped) + Hot water source (No access)	1/2+1/2	1
Number of people in home (Less than 3) + Number of bedrooms in home (3 or more) + pets in home	1/3+1/3+1/3	1
Number of people sharing a bathroom (Less than 3) + Type of toilet facility (Bucket, pit latrine or no facility)	1/2+1/2	1
Raw beef consumption (once per year or more)	1	1
Unpasteurized milk consumption (once per year or more)	1	1
Helminth infection (not exposed) + Treatment for helminths	1/2+1/2	1
Community type + Donkey/horse/sheep/cow on property	1/2+1/2	1
Passive Cigarette smoke exposure (exposure)	1	1
**Total Possible Score**	**8**	**8**

**Table 3:** Score analysis for Method B.

Environmental Risk Factor Combination	Weighting for each variable	Total score for subgroup
Source of drinking water (Not piped) + Hot water (No access) + Raw beef consumption (once per year or more)	1/3+1/3+1/3	1
Number of people in a home (Less than 3) + Number of bedrooms in a home (3 or more) + Number of people sharing a bathroom (Less than 3) + Type of toilet facility (Bucket, pit latrine or no facility) + pets in home	1/5+1/5+1/5+1/5+1/5	1
Community type (Suburban or Urban) + Donkey/horse/sheep/cow living permanently on property (no exposure) + Helminth infection (no exposure) + treatment for helminths	1/4+1/4+1/4+1/4	1
Unpasteurized milk consumption (once per year or more)	1	1
Passive Cigarette smoke exposure (exposure)	1	1
**Total Possible Score**	**5**	**5**

It must be noted that for both Method A and Method B, the exposure variables ‘never having consumed unpasteurized milk’ and ‘passive cigarette smoke exposure’ were not combined with other exposure variables and their individual score remained equal to the value of one. As this was a post-hoc analysis, this weighting methodology was decided based on the following: 1) a significant risk association was identified for unpasteurized milk during all 3 age intervals, including an independent risk association for 6–10 and 11–18 years; and 2) a significant association was identified for passive cigarette smoke exposure during 2 age intervals, including of an independent risk association for 11–18 years [4].

Method A and Method B consisted of individual subgroups, and the lowest possible variable weighting within any subgroup was equal to 1/5, thus a subgroup consisted of no more than 5 exposure variables. Since the ‘score value’ for every subgroup was equal to the value of one, the total possible score for Method A and Method B was equivalent to the total number of subgroups contained within that group, respectively. For Method A, the total possible score was 8, and for Method B, the total possible score was five. A score analysis was performed based on these premises. The subgroups within Method A and Method B, respectively, were statistically analysed based on their single (i.e. unpasteurized milk) or pooled weighted score.

Comparisons were performed between the cases and the controls using these predefined approaches (Method A and Method B), and the score analysis for environmental risk factors was performed over the 3 childhood age intervals (0–5, 6–10 and 11–18 years). For each analysis (0–5, 6–10 and 11–18 years), score results have been represented as minimum, maximum, mean and median values for the case and control groups. The Odds Ratios (OR) and 95%CIs represents the significance of the difference between the two groups, as well as the difference in proportion with regard to number of exposures based on the score.

### Statistical analysis

Demographic data for the cases and controls is presented as frequencies (percentages) for the childhood infection and as medians, and as medians (interquartile range (IQR)) and mean (standard deviation (SD)) for the environmental exposure variables (environmental risk factors). The score analysis was conducted for the environmental exposures over the three age intervals using logistics regression.

### Ethics statement

Ethical clearance was granted by the Senate Research Ethics Committee of the University of the Western Cape (Reg. no. 11/3/16), the Human Research Ethics Committee of the University of Cape Town (Ref. no. 122/2011) and the Provincial Department of Health. All participants gave written informed consent.

## Results

### Score analysis for ‘Method 0’: Environmental risk factors during the 3 age intervals

The results of the score analysis for Method 0, evaluating environmental risk factor exposure in 3 age groups (0–5 years, 6–10 years and 11–18 years) are shown in Table 4. In this model, all exposures were equal to the value of one, thus the total possible score was 14 (Table 1).

**Table 4:** Score analysis for environmental risk factors during the 3 age intervals.

**0–5 Age group**				
	**Cases**	**Control**	**Overall**	**OR (95% CI)**
**Mean**	4.39	4.71	4.56	0.92 (0.83, 1.02)
**Median, IQR**	4 (3.0,5.0)	4 (3.0,6.0)	4 (3.0, 6.0)	
**SD**	1.93	1.98	1.96	
**Min**	0	0	0	
**Max**	10	12	12	
**6–10 Age group**				
**Mean**	4.39	4.6	4.5	0.94 (0.80, 1.02)
**Median, IQR**	4 (3.0, 6.0)	4 (3.0, 6.0)	4 (3.0, 6.0)	
**SD**	1.80	1.92	1.86	
**Min**	0	0	0	
**Max**	9	11	11	
**11–18 Age group**				
**Mean**	3.77	4.02	3.90	0.90 (0.80, 1.02)
**Median, IQR**	4 (3.0, 5.0)	4 (3.0, 5.0)	4 (3.0, 5.0)	
**SD**	1.54	1.65	1.60	
**Min**	0	1	0	
**Max**	9	9	9	

#### 0–5 years

During the age interval 0–5 years, the mean and median scores for the case and control group were [4.39 (SD ± 1.93) vs 4.71 (SD ± 1.98); and 4.0 (IQR 3.0–5.0) vs 4.0 (IQR 3.0–6.0)], respectively. Both groups had a minimum score of zero, whereas cases had a maximum score of 10 and controls had a maximum score of 12. There was no significant difference in the exposure scores between the case and control groups (OR = 0.92; 95% CI, 0.83–1.02).

#### 6–10 years

During the age interval 6–10 years, the mean and median scores for the case and control group were [4.39 (SD ± 1.80) vs 4.60 (SD ± 1.92); and 4.0 (IQR 3.0–6.0) vs 4.0 (IQR 3.0–6.0)], respectively. Both groups had a minimum score of zero, whereas cases had a maximum score of 9 and controls had a maximum score of 11. There was no significant difference in the exposure scores between the case and control groups (OR = 0.94; 95% CI, 0.80–1.02).

#### 11–18 years

During the age interval 11–18 years, the mean and median scores for the case and control group were [3.77 (SD ± 1.54) vs 4.02 (SD ± 1.65); and 4.0 (IQR 3.0–5.0) vs 4.0 (IQR 3.0–5.0)], respectively. The minimum and maximum scores for cases were 0 and 9, respectively, whereas the minimum and maximum scores for controls were 1 and 9, respectively. There was no significant difference in the exposure scores between the case and control groups (OR = 0.90; 95% CI, 0.80–1.02).

### Score analysis for environmental risk factors during the three age intervals

#### Method A

The results of the score analysis evaluating environmental risk factor exposure in 3 age groups (0–5 years, 6–10 years and 11–18 years) for Method A are shown in Table 5. The maximum possible score for Method A was 8.

**Table 5:** Score analysis for environmental exposures during 3 age intervals; Group A.

**0–5 years**				
	**Cases**	**Control**	**Overall**	**OR (95% CI)**
**Mean**	2.08	3.58	3.40	**0.74 (0.62, 0.90)**
**Median, IQR**	1.99 (1.33, 2.66)	2.16 (1.66, 3.16)	2.16 (1.49, 2.83)	
**SD**	0.98	1.12	1.07	
**Min**	0	0	0	
**Max**	5.16	6.49	6.49	
**6–10 years**				
**Mean**	1.30	1.64	1.48	**0.67 (0.53, 0.83)**
**Median, IQR**	1.5 (0.5, 2.0)	1.5 (1.0, 2.5)	1.5 (0.5, 2.0)	
**SD**	0.79	1.03	0.94	
**Min**	0	0	0	
**Max**	3.5	6	6	
**11–18 years**				
**Mean**	1.81	2.08	1.95	**0.72 (0.58, 0.90)**
**Median, IQR**	1.66 (1.16, 2.33)	1.83 (1.33, 2.08)	1.83 (1.33, 2.49)	
**SD**	0.85	0.98	0.93	
**Min**	0	0.33	0	
**Max**	4.66	5.16	5.16	

#### 0–5 years

During the age interval 0–5 years, the mean and median scores for the case and control group were [2.08 (SD ± 0.98) vs 3.58 (SD ± 1.12); and 1.99 (IQR 1.33–2.66) vs 2.16 (IQR 1.66–3.16)], respectively. Both groups had a minimum score of zero, whereas cases had a maximum score of 5.16 and controls had a maximum score of 6.46. There was a significant difference in exposure scores between the case and control groups (OR = 0.74; 95% CI, 0.62–0.92), thus indicating that cases had 26% less exposure during this age interval when compared to the controls.

#### 6–10 years

During the age interval 6–10 years, the mean and median scores for the case and control group were [1.30 (SD ± 0.79) vs 1.64 (SD ± 1.03); and 1.50 (IQR 0.5–2.0) vs 1.50 (IQR 1.0–2.5)], respectively. Both groups had a minimum score of zero, whereas cases had a maximum score of 3.5 and controls had a maximum score of 6. There was a significant difference in exposure scores between the case and control groups (OR = 0.67; 95% CI, 0.53–0.83), thus indicating that cases had 33% less exposure during this age interval when compared to the controls.

#### 11–18 years

During the age interval 11–18 years, the mean and median scores for the case and control group were [1.81 (SD ± 0.85) vs 2.08 (SD ± 0.98); and 1.66 (IQR 1.16–2.33) vs 1.83(IQR 1.33–2.08)], respectively. The minimum and maximum scores for cases were 0 and 4.66, respectively, whereas the minimum and maximum scores for controls were 0.33 and 5.16, respectively. There was a significant difference in exposure scores between the case and control groups (OR = 0.72; 95% CI, 0.58–0.90), thus indicating that cases had 28% less exposure during this age interval when compared to the controls.

#### Method B

The results of the score analysis evaluating environmental risk factor exposure in 3 age groups (0–5 years, 6–10 years and 11–18 years) for Method B are shown in Table 6.

**Table 6:** Score analysis for environmental exposures during 3 age intervals; Group B.

**0–5 years**				
	**Cases**	**Control**	**Overall**	**OR (95% CI)**
**Mean**	1.20	1.47	1.34	**0.58 (0.43, 0.77)**
**Median, IQR**	1.10 (0.73, 1.6)	1.38 (0.85, 1.98)	1.22 (0.8, 1.78)	
**SD**	0.63	0.77	0.72	
**Min**	0	0	0	
**Max**	3.13	3.93	3.93	
**6–10 years**				
**Mean**	1.22	1.48	1.36	**0.58 (0.43, 0.78)**
**Median, IQR**	1.19 (0.73, 1.60)	1.33 (0.81, 1.98)	1.23 (0.76, 1.8)	
**SD**	0.6	0.78	0.71	
**Min**	0	0	0	
**Max**	3.06	4.26	4.26	
**11–18 years**				
**Mean**	1.08	1.31	1.21	**0.59 (0.43, 0.79)**
**Median, IQR**	0.93 (0.64, 1.41)	1.19 (0.73, 1.8)	0.98 (0.73, 1.65)	
**SD**	0.60	0.71	0.67	
**Min**	0	0.2	0	
**Max**	3.13	3.63	3.68	

#### 0–5 years

During the age interval 0–5 years, the mean and median scores for the case and control group were [1.20 (SD ± 0.63) vs 1.47 (SD ± 0.77); and 1.10 (IQR 0.73–1.60) vs 1.38 (IQR 0.85–1.98)], respectively. Both groups had a minimum score of zero, whereas cases had a maximum score of 3.13 and controls had a maximum score of 3.93. The maximum possible score for Method B was 5. There was a significant difference in exposure scores between the case and control groups (OR = 0.72; 95% CI, 0.58–0.90), thus indicating that cases had 42% less exposure during this age interval when compared to the controls.

#### 6–10 years

During the age interval 6–10 years, the mean and median scores for the case and control group were [1.22 (SD ± 0.60) vs 1.33 (SD ± 0.78); and 1.19 (IQR 0.73–1.60) vs 1.33 (IQR 0.81–1.98)], respectively. Both groups had a minimum score of zero, whereas cases had a maximum score of 3.06 and controls had a maximum score of 4.26. There was a significant difference in exposure scores between the case and control groups (OR = 0.58; 95% CI, 0.43–0.78), thus indicating that cases had 42% less exposure during this age interval when compared to the controls.

#### 11–18 years

During the age interval 11–18 years, the mean and median scores for the case and control group were [1.08 (SD ± 0.60) vs 1.31 (SD ± 0.71); and 0.93 (IQR 0.64–1.41) vs 1.19 (IQR 0.73–1.80)], respectively. The minimum and maximum scores for cases were 0 and 3.13, respectively, whereas the minimum and maximum scores for controls were 0.2 and 3.63, respectively. There was a significant difference in exposure scores between the case and control groups (OR = 0.59; 95% CI, 0.43–0.79), thus indicating that cases had 41% less exposure during this age interval when compared to the controls.

## Discussion

The enteric flora is comprised of numerous microorganisms [31] and the history of CD is paved with publications hypothesizing various environmental agents in the aetiology of CD [20, 32, 33]. However, studies evaluating exposures during childhood have largely focused on the effect of an individual environmental exposure without accounting for potential confounding interactions both between variables and over time, likely limiting the true validity of what is being measured. Using a subset of previously collected data, the present study conducted a post-hoc score analysis to investigate this relationship in an attempt to further delineate the paradigm of the hygiene hypothesis.

Results of the score analysis (Method 0) for the environmental exposures during 3 age intervals (0–5, 6–10 and 11–18 years) revealed no significant difference between the case and control groups (Table 4). All exposure variables were equal to a value of one and in turn, frequency of exposure was based on the individual effect of each variable, thus implying that in most cases, the capacity of a single variable to induce dysbiosis within the gut microbiome is limited. By contrast, a significant case-control difference was observed in all age intervals evaluated for both Method A and Method B, with cases having significantly lower exposure scores (approximately 30% and 40% lower, respectively), when compared with that of controls. In support of the hygiene hypothesis, these findings implicate the complex role in which multiple microbial-based environmental exposures function in the development of the intestinal immune system. The latter is further portrayed by the change in both the mean values and CIs observed between Method A and Method B in that, the difference in mean values between the cases and controls in Method A is consistently larger compared to the difference in mean values for cases and controls in Method B, as well as that narrower CIs are consistently observed for Method A when compared with Method B, during all 3 age intervals.

One important caveat underlying the development of the gut microbiome is CD susceptibility mutations [20, 34]. While it is recognized that the gut microbiome is profoundly shaped by the microbial environment, there is now evidence that the converse is also true [35–37]. For instance, it has been shown that the intricate array of pattern recognition receptors (PRR)s, including the vitamin D receptor (VDR), which equip the immune system to recognize microbial molecular patterns, have an impressive effect on both the diversity and functionality of the gut microbiota [38]. Therefore, genetic alterations in these PPRs may influence this bidirectional interaction, in which immune activity can either suppress or promote pathogenic microbial blooms, in turn, affecting homeostasis of host intestinal immunity and the convergence of disease susceptibility. The NOD-like or nucleotide oligomerization domain receptors (encoded by NOD2 gene) is a PRR that recognizes intracellular bacterial products and variants in the CARD15/NOD2/IBD1 locus are associated with the development and phenotypic patterns of CD [39]. The likelihood of a NOD2-dysregulating microbial community in immune-mediated disorders, such as CD is strengthened by data from recent NOD2-/- mice [40], in which unregulated inflammation of the gut results taxonomic shifts in bacterial phyla characteristic of the change in the microbiome during inflammation, and similar to those described in IBD patients compared with healthy controls.

Findings from the score analysis performed in the present study provide insight into the ‘compound’ effects from environmental risk factors in the pathogenesis of CD. This has important implications for future IBD-related studies as it demonstrates the importance of accounting for environment as a ‘whole’ during epidemiological research, as opposed to the impact of individual factors. Indeed, it is likely that certain environmental risk factors may hold a greater ‘weight’ with regard to their effect on the gut microbiota in disease pathogenesis, particularly in context to timing of exposure during childhood.

## Conclusion

The findings from this present study are in line with the hygiene hypothesis, and demonstrate the complex role in which numerous microbial-based environmental exposures together with CD susceptibility genes function in the development of the gastro-intestinal immune system. Furthermore, the score analysis provides insight into the ‘compound’ effect from environmental risk factors in the pathogenesis of CD in which certain environmental exposures during childhood have greater impact with regard to their effect on the gut microbiota in disease pathogenesis, together with timing of exposure.

## References

[1] Ng SC, Bernstein CN, Vatn MH, Lakatos PL, Loftus EV Jr, Tysk C, et al Epidemiology and Natural History Task Force of the International Organization of Inflammatory Bowel Disease (IOIBD): Geographical variability and environmental risk factors in inflammatory bowel disease. Gut 2013, 62(4):630–649. 23335431 10.1136/gutjnl-2012-303661

[2] Molodecky NA, Soon S, Rabi DM, Ghali WA, Ferris M, Chernoff G, et al Increasing incidence and prevalence of the inflammatory bowel diseases with time, based on systematic review. Gastroenterol 2012, 142(1):46–54. e42. 10.1053/j.gastro.2011.10.001 22001864

[3] Danese S, Sans M, Fiocchi C. Inflammatory bowel disease: the role of environmental factors. Autoimmun Rev 2004, 3(5):394–400. 10.1016/j.autrev.2004.03.002 15288007

[4] Basson A, Swart R, Jordaan E, Mazinu M, Watermeyer G. The association between childhood environmental exposures and the subsequent development of Crohn's disease in the Western Cape, South Africa. PloS one 2014, 9(12):e115492 10.1371/journal.pone.0115492 25514591 PMC4267820

[5] Bernstein CN, Rawsthorne P, Cheang M, Blanchard JF. A population-based case control study of potential risk factors for IBD. Am J Gastroenterol 2006, 101(5):993–1002. 10.1111/j.1572-0241.2006.00381.x 16696783

[6] Castiglione F, Diaferia M, Morace F, Labianca O, Meucci C, Cuomo A, et al Risk factors for inflammatory bowel diseases according to the "hygiene hypothesis": a case-control, multi-centre, prospective study in Southern Italy. J Crohns Colitis 2012, 6(3):324–329. 10.1016/j.crohns.2011.09.003 22405169

[7] Gearry RB, Richardson AK, Frampton CM, Dodgshun AJ, Barclay ML. Population-based cases control study of inflammatory bowel disease risk factors. J Gastroenterol Hepatol 2010, 25(2):325–333. 10.1111/j.1440-1746.2009.06140.x 20074146

[8] Guo AY, Stevens BW, Wilson RG, Russell CN, Cohen MA, Sturgeon HC, et al Early life environment and natural history of inflammatory bowel diseases. BMC Gastroenterol 2014, 14:216-014-0216-8. 10.1186/s12876-014-0216-8 PMC4300207 25510175

[9] Hampe J, Heymann K, Krawczak M, Schreiber S. Association of inflammatory bowel disease with indicators for childhood antigen and infection exposure. Int J Colorectal Dis 2003, 18(5):413–417. 10.1007/s00384-003-0484-1 12687394

[10] Han DY, Fraser AG, Dryland P, Ferguson LR. Environmental factors in the development of chronic inflammation: A case-control study on risk factors for Crohn's disease within New Zealand. Mutat Res Fund Mol Mech Mut 2010, 690(1):116–122. 10.1016/j.mrfmmm.2009.09.002 19751746

[11] Hansen TS, Jess T, Vind I, Elkjaer M, Nielsen MF, Gamborg M, et al Environmental factors in inflammatory bowel disease: a case-control study based on a Danish inception cohort. J Crohns Colitis 2011, 5(6):577–584. 10.1016/j.crohns.2011.05.010 22115378

[12] Hildebrand H, Malmborg P, Askling J, Ekbom A, Montgomery SM. Early-life exposures associated with antibiotic use and risk of subsequent Crohn's disease. Scand J Gastroenterol 2008, 43(8):961–966. 19086166 10.1080/00365520801971736

[13] Hlavaty T, Toth J, Koller T, Krajcovicova A, Oravcova S, Zelinkova Z, et al Smoking, breastfeeding, physical inactivity, contact with animals, and size of the family influence the risk of inflammatory bowel disease: A Slovak case-control study. United European Gastroenterol J 2013, 1(2):109–119. 10.1177/2050640613478011 24917948 PMC4040732

[14] López-Serrano P, Pérez-Calle JL, Pérez-Fernández MT, Fernández-Font JM, Boixeda de Miguel D, Fernández-Rodríguez CM. Environmental risk factors in inflammatory bowel diseases. Investigating the hygiene hypothesis: a Spanish case-control study. Scand J Gastroenterol 2010, 45(12):1464–1471. 10.3109/00365521.2010.510575 20704469

[15] Foster A, Jacobson K. Changing incidence of inflammatory bowel disease: environmental influences and lessons learnt from the South Asian population. Front Paediatr 2013, 1:34. 10.3389/fped.2013.00034 PMC3864265 24400280

[16] Ananthakrishnan AN, Huang H, Nguyen DD, Sauk J, Yajnik V, Xavier RJ. Differential effect of genetic burden on disease phenotypes in Crohn's disease and ulcerative colitis: analysis of a North American cohort. Am J Gastroenterol 2014, 109(3):395–400. 10.1038/ajg.2013.464 24419484 PMC4225079

[17] Korzenik JR. Past and current theories of aetiology of IBD: toothpaste, worms, and refrigerators. J Clin Gastroenterol 2005, 39(4):S59–S65. 15758661 10.1097/01.mcg.0000155553.28348.fc

[18] Chamberlin WM, Naser SA. Integrating theories of the aetiology of Crohn's disease. On the aetiology of Crohn's disease: questioning the hypotheses. Med Sci Monit 2006, 12(2):RA27–33. 16449960

[19] Koloski NA, Bret L, Radford-Smith G. Hygiene hypothesis in inflammatory bowel disease: a critical review of the literature. World journal of gastroenterology 2008, 14(2):165 18186549 10.3748/wjg.14.165 PMC2675108

[20] Sartor RB. Mechanisms of disease: pathogenesis of Crohn's disease and ulcerative colitis. Nat Clin Pract Gastroenterol Hepatol 2006, 3(7):390–407. 10.1038/ncpgasthep0528 16819502

[21] Guarner F, Bourdet-Sicard R, Brandtzaeg P, Gill HS, McGuirk P, Van Eden W, et al Mechanisms of disease: the hygiene hypothesis revisited. Nature Clinical Practice Gastroenterology & Hepatology 2006, 3(5):275–284. 10.1038/ncpgasthep0471 16673007

[22] Hugot J, Zaccaria I, Cavanaugh J, Yang H, Vermeire S, Lappalainen M, et al Prevalence of CARD15/NOD2 mutations in Caucasian healthy people. Am J Gastroenterol 2007, 102(6):1259–1267. 10.1111/j.1572-0241.2007.01149.x 17319929

[23] Loftus EV Jr. Management of extraintestinal manifestations and other complications of inflammatory bowel disease. Curr Gastroenterol Rep 2004, 6(6):506–513. 15527681 10.1007/s11894-004-0073-7

[24] Silverberg MS, Satsangi J, Ahmad T, Arnott ID, Bernstein CN, Brant SR, et al Toward an integrated clinical, molecular and serological classification of inflammatory bowel disease: Report of a Working Party of the 2005 Montreal World Congress of Gastroenterology. Canadian Journal of Gastroenterology and Hepatology 2005, 19(Suppl A):5A–36A. 10.1155/2005/269076 16151544

[25] Strisciuglio C, Auricchio R, Martinelli M, Staiano A, Giugliano FP, Andreozzi M, et al Autophagy genes variants and paediatric Crohn's disease phenotype: A single-centre experience. Digestive and Liver Disease 2014, 46(6):512–517. 24656308 10.1016/j.dld.2014.02.016

[26] Karatzas PS, Gazouli M, Safioleas M, Mantzaris GJ. DNA methylation changes in inflammatory bowel disease. Annals of Gastroenterology 2014, 27(2):125 24733658 PMC3982627

[27] Rogler G, Vavricka S. Exposome in IBD: recent insights in environmental factors that influence the onset and course of IBD. Inflamm Bowel Dis 2015, 21(2):400–408. 10.1097/MIB.0000000000000229 25358064

[28] Strisciuglio C, Giugliano FP, Martinelli M, Cenni S, Greco L, Staiano A, et al Impact of Environmental and Familial Factors in a Cohort of Paediatric Patients with Inflammatory Bowel Disease. J Paediatr Gastroenterol Nutr 2016, 10.1097/MPG.0000000000001297 27306105

[29] Basson A, Swart R, Jordaan E, Mazinu M, Watermeyer G. The association between race and Crohn's disease phenotype in the Western Cape population of South Africa, defined by the Montreal Classification System. PloS one 2014, 9(8):e104859 10.1371/journal.pone.0104859 25118187 PMC4130615

[30] Epstein D, Watermeyer G, Kirsch R. Review article: the diagnosis and management of Crohn’s disease in populations with high-risk rates for tuberculosis. Aliment Pharmacol Ther 2007, 25(12):1373–1388. 10.1111/j.1365-2036.2007.03332.x 17539977

[31] Johnson CL, Versalovic J. The human microbiome and its potential importance to paediatrics. Paediatrics 2012, 129(5):950–960. 10.1542/peds.2011-2736 PMC3340594 22473366

[32] De Hertogh G, Aerssens J, Geboes KP, Geboes K. Evidence for the involvement of infectious agents in the pathogenesis of Crohn's disease. World J Gastroenterol 2008, 14(6):845 18240341 10.3748/wjg.14.845 PMC2687051

[33] Rosenfeld G, Bressler B. Mycobacterium avium paratuberculosis and the aetiology of Crohn’s disease: a review of the controversy from the clinician’s perspective. Can J Gastroenterol 2010, 24(10):619–624. 21037992 10.1155/2010/698362 PMC2975476

[34] Strober W, Kitani A, Fuss I, Asano N, Watanabe T. The molecular basis of NOD2 susceptibility mutations in Crohn's disease. Mucosal Immunol 2008, 1:S5–S9. 10.1038/mi.2008.42 19079230 PMC3665012

[35] Kostic AD, Xavier RJ, Gevers D. The microbiome in inflammatory bowel disease: current status and the future ahead. Gastroenterol 2014, 146(6):1489–1499. 10.1053/j.gastro.2014.02.009 PMC4034132 24560869

[36] Knights D, Lassen KG, Xavier RJ. Advances in inflammatory bowel disease pathogenesis: linking host genetics and the microbiome. Gut 2013, 62(10):1505–1510. 10.1136/gutjnl-2012-303954 24037875 PMC3822528

[37] Leone V, Chang EB, Devkota S. Diet, microbes, and host genetics: the perfect storm in inflammatory bowel diseases. J Gastroenterol 2013, 48(3):315–321. 10.1007/s00535-013-0777-2 23475322 PMC3698420

[38] Kamada N, Seo S, Chen GY, Núñez G. Role of the gut microbiota in immunity and inflammatory disease. Nat Rev Immunol 2013, 13(5):321–335. 10.1038/nri3430 23618829

[39] Biswas A, Petnicki-Ocwieja T, Kobayashi KS. Nod2: a key regulator linking microbiota to intestinal mucosal immunity. J Mol Med 2012, 90(1):15–24. 10.1007/s00109-011-0802-y 21861185 PMC3263373

[40] Couturier-Maillard A, Secher T, Rehman A, Normand S, De Arcangelis A, Haesler R, et al NOD2-mediated dysbiosis predisposes mice to transmissible colitis and colorectal cancer. J Clin Invest 2013, 123(2):700–7 10.1172/JCI62236 23281400 PMC3561825

