# Correction: The association between environmental exposures during childhood and the subsequent development of Crohn's disease: A score analysis approach

**DOI:** 10.1371/journal.pone.0190417

**Published:** 2017-12-21

**Authors:** Victor Tinashe Sabe, Abigail Raffner Basson, Esme Jordaan, Mikateko Mazinu, Gillian Watermeyer

Dr. Gillian Watermeyer is not included in the author byline. She should be listed as the fifth author and affiliated with the Department of Gastroenterology, Groote Schuur Hospital, Cape Town, Western Cape, South Africa and the Department of Medicine, University of Cape Town, Cape Town, Western Cape, South Africa. The contributions of this author are as follows: conceptualization, data curation, funding acquisition, resources, supervision, validation.

The correct citation is: Sabe VT, Basson AR, Jordaan E, Mazinu M, Watermeyer G (2017) The association between environmental exposures during childhood and the subsequent development of Crohn’s disease: A score analysis approach. PLoS ONE12(2): e0171742. https://doi.org/10.1371/journal.pone.0171742

There are errors in the author affiliations. The affiliations should appear as shown here:

Victor Tinashe Sabe^1^, Abigail Raffner Basson^2^, Esme Jordaan^3^, Mikateko Mazinu^4^, Gillian Watermeyer^5, 6^

**1** Medical BioScience Department, University of the Western Cape, Bellville, Western Cape, South Africa, **2** Digestive Health Research Institute, Case Western Reserve University, Cleveland, Ohio, United States of America, **3** Statistics and Population Studies Department, University of the Western Cape, Bellville, Western Cape, South Africa, **4** Biostatistics Unit, Medical Research Council of South Africa, Parow, Western Cape, South Africa, **5** Department of Gastroenterology, Groote Schuur Hospital, Cape Town, Western Cape, South Africa, **6** Department of Medicine, University of Cape Town, Cape Town, Western Cape, South Africa.
